# Factors affecting subsequent dose of COVID-19 vaccine uptake based on BASNEF model among older adults

**DOI:** 10.1186/s12879-023-08903-8

**Published:** 2024-01-02

**Authors:** Majid Barati, Hanieh Jormand, Salman Khazaei, Saeed Bashirian, Mohadeseh Sadri, Maryam Afshari

**Affiliations:** 1 Social Determinants of Health Research Center, Hamadan University of Medical Sciences and Department of Public Health, Asadabad School of Medical sciences, Asadabad, Iran, Hamadan, IR Iran; 2 Urology and Nephrology Research Center, Hamadan University of Medical Sciences, Hamadan, IR Iran; 3 Department of Epidemiology, School of Public and Social Determinants of Health Research Center, Hamadan University of Medical Sciences, Hamadan, IR Iran; 4 Department of Public Health, School of Public Health and Social Determinants of Health Research Center, Hamadan University of Medical Sciences, Hamadan, IR Iran; 5 Iranian Research Center on Aging, University of Social Walfare and Rehablitation Sciences, Tehran, IR Iran

**Keywords:** Intention, Immunosenescence, Aging, COVID-19 vaccines

## Abstract

**Background:**

Vaccination is a primary prevention approach to preventing disease by disconnecting the transmission chain. The current study utilized a BASNEF model framework to identify factors influencing subsequent doses of COVID-19 vaccination among older adults.

**Methods:**

This cross-sectional study was performed in the west of Iran in May 2022. The participants were selected via multi-stage sampling. Finally, 1120 participants contributed to the present study. The questionnaire consisted of three sections: a) Socio-demographic characteristics, b) cognitive impairments tests, and c) Questionnaire about the subsequent dose of COVID-19 vaccine uptake based on the BASNEF model. Data were analyzed using the software IBM AMOS-20 and SPSS-23 via one-way analysis of variance (ANOVA) and independent sample T-tests were used, too. The significance level of statistical tests was regarded as less than 0.05.

**Results:**

The presented results of analyzing 50% of the variance of vaccination intention as the dependent variable (R square = 0.497) and 10% of the behavior variance as the dependent variable (R square = 0.104) can be explained based on the BASNEF model. The enabling factors (β = 0.636, *p* < 0.001) and the intention (β = 0.322, *p* < 0.001) were important factors for subsequent doses of COVID-19 vaccine uptake in older adults.

**Conclusion:**

So, planning and implementing promotional intervention programs for older people (over 65; 80), females, illiterate, widows and divorced, good economic status, and urban areas is essential. It seems that enabling factors such as free vaccinations, vaccination inaccessible places such as public social security agencies, social supports such as involvement of the government and physicians, and improving information by the medium or knowledge-sharing experience, which can be further used to enhance the acceptance of subsequent doses of COVID-19 uptake in older adults.

## Introduction

Vaccination is a primary prevention approach to disease prevention by disconnecting the transmission chain. In addition, Older adults are vulnerable to severe morbidity and mortality from communicable illnesses. Older adults may be insufficient immunocompetent, which goes together with aging [1]. WHO's Strategic Advisory Group of Experts on Immunization (SAGE), in March 2023, updated its recommendations on primary series vaccination (two doses of any WHO EUL vaccine) and the need for booster doses for the current context. The high-priority groups are populations with the most significant risk of severe disease and death. They include the oldest and older adults with multiple significant comorbidities. The high-priority group should be prioritized for the primary series vaccines and first and additional booster doses. The other boosters should be administered 6 or 12 months (depending on your risk category) after the last dose, depending on age and immunocompromising conditions [2]. Overall, decision‐making by individual ages is different, as, in younger adults, it is more rapid than in older generations [3, 4], especially in health care services [5]. Also, another challenge in dealing with an aging population is the change of factors affecting the willingness to be vaccinated [6].

However, there is vaccine hesitancy with delays in accepting or refusing vaccines despite available services, as even the World Health Organization (WHO) considered it a global health threat in 2019 [1, 3]. However, public policy workers are facing challenges in immunizing this susceptible group [4].

Evidence showed that health behavior factors such as subjective norms, cues to action, perceived barriers, behavioral beliefs, knowledge, and information sources accompanied by demographic factors such as age, sex, and living with others influence primary series vaccination and influenza vaccination behavior among older adults [5]. So, all constructs mentioned were of the health belief model (HBM), and some concepts of the theory of reasoned action (TRA) or intentional model constructs. While the intention factor is an important factor in the consequences of behavior concerning a gap" between intentions and behavior, it was completed with psychological variables that might be able to "bridge" the intention–behavior gap [7]. One reason for failing to improve healthy behaviors is the lack of consideration of the factors affecting behaviors and psychosocial models as specific intellectual frameworks [8, 9]. On the other hand, evidence proved that enabling factors such as income, urbanization of the resistant area, and social support are potentially associated with the patterns of repeated influenza vaccination in older adults [10]. Also, evidence has highlighted the strategies that are required for COVID-19 vaccination acceptance, such as massive manufacturing and distribution of millions or trillions of doses to the global population, localized larger trust-building and vaccine-delivery-system-strengthening actions are necessary to involve the community, and above all, resolve fears and misconceptions [11].

**Fig. 1:**
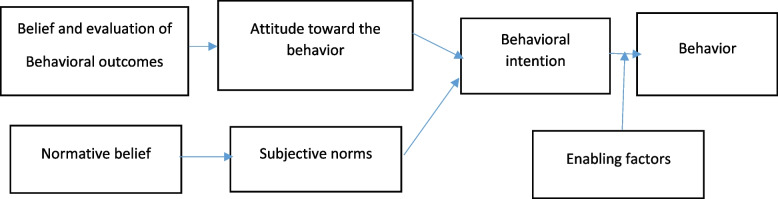
The framework of the BASNEF model (beliefs, attitudes, subjective norms and enabling factors) [17]

So, the BASNEF model is based on Fishbein's theory regarding attitude, behavioral intention, subjective norms, and enabling factors such as the skill of performing a behavior, time, and costs [8, 9, 12] (Fig. 1). The BASNEF model was considered to apply in the present study and, considering this model is applied to many other studies to determine the effective factors that lead to healthy behaviors concerning norms, social pressures, and attitudes towards a behavior [12–15]. Also, the results of the study showed that the main reasons for the reinforcement of primary COVID-19 vaccine refusals were fear of side effects (41.2%) and lack of confidence in vaccine effectiveness (15.1%) in LMICs [16]. Therefore, in the present study, the beliefs, attitudes, subjective norms, and enabling factors in the BASNEF model were used to identify the effective factors affecting the subsequent dose of COVID-19 vaccine uptake among older adults.

## Methods

### Statements

The Ethical Committee approved the protocols of the present study at Hamadan University of Medical Science (IR.UMSHA.REC.1400.174). Informed consent was obtained from all participants; they were informed about the confidentiality of the information, the project's purpose, and their voluntary participation in the study. All methods were performed based on relevant guidelines and regulations. The confidentiality of the participants' information was also assured. Informed consent was obtained from all the contributors.

Hence, this research hypothesizes:

- *H1*. The attitude will have a positive and significant effect on their vaccine intention.
- *H2*. The Subjective norms will positively and significantly affect their vaccine intention.
- *H3*. The enabling factors will positively and significantly affect COVID-19 vaccine intention.
- *H4*. The COVID-19 vaccine intention has a positive and significant effect on behavior.
- H5: The intention moderates the relation between attitude and behavior.
- H6: The intention moderates the relation between subjective norms and behavior.
- H7: The intention moderates the relation between enabling factors and behavior.

**Fig. 2:**
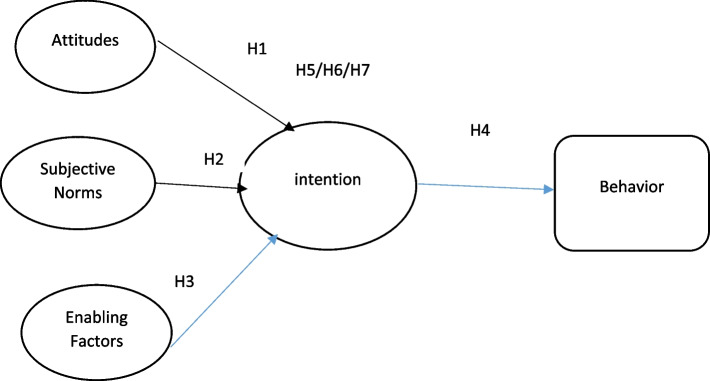
The conceptual framework of factors affecting subsequent doses of COVID-19 vaccination

Figure 2 illustrates the hypothesized relationships of this research.

### Design and participants

This cross-sectional study was performed in the west of Iran, including Hamadan, Kermanshah, Kordestan, and Lorestan Provinces, in May 2022. The required sample size was estimated at 1,142 cases, assuming that 90% of older adults in the pilot study were done to estimate subsequent doses, considering a 95% confidence interval, a margin of error of 0.02, and the design effect equal to 2. Finally, 1,120 subjects were included in the study. The participants were selected via multi-stage sampling (sequence of stratified- simple random sampling) with proportional size weights. Firstly, cases were stratified to public social security agencies [18]. After that, we assigned them a sample size proportional to the population size of different age categories. We received the mobile number of older adults from the relevant manager for each age category according to the allocated sample size via random sampling. A new person was randomly replaced by those who did not respond. But the response rate was 98%. Finally, 1120 participants contributed to the present study. The inclusion criteria were as follows: 1) regular appearance in the retirement center, and b) willingness to participate in the survey, age > 60 years, ability to complete the study, ability to speak and read in Farsi, having a history of COVID-19 vaccine uptake at least twice, and filling out a consent form to participate in the study.

### Data collection

All methods were performed following relevant guidelines and regulations (STROBE). Written informed consent was obtained from all patients; they were informed about the confidentiality of the information and the project's purpose. Only if they would like to be enrolled in the study. The Ethics Committee approved this study with all consent processes at Hamadan University of Medical Sciences.

### Measurements

The data-gathering tools were a mix of a researcher-designed questionnaire (part A and C) and an existing questionnaire (part B) as a self-statement tool. The questionnaire used in this study consisted of three sections: A) socio-demographic characteristics including age, gender, educational status, number of families, economy and marital status, the region and area of residence, the chronic condition of disease, had a positive test for COVID-19 infection, the source for Coronavirus information, and the influenza vaccine uptake.

B) The cognitive impairment tests include 6-CIT, a simple first-level cognitive screening tool composed of six questions: three about orientation, one about memory, and two calculations. The sum scores from 0 (cognitively intact) to 28 (maximum impairment). In its validation, the 7.8 cut-off offered optimal sensitivity and specificity [19–21].

Instrumental Activities of Daily Living (IADL) are used to assess the autonomy in performing more complex tasks such as telephone use or handling finances. It contains eight items. The score ranges from 8 (completely autonomous) to 0 (entirely dependent) [20]. In the present study, the Persian version of IADL in older adults was used and applied in studies; although the CVR was reported to be more than 0.82, the sensitivity and specificity of IADL were 0.96. Cronbach's alpha and ICC were more than 0.75. [22, 23]. It contained seven items. The score ranges of the pension version were 0–14, with 0–6 (entirely dependent), 7–10 (a little dependent), and (11–14) (completely autonomous).

C) Questionnaire about the booster dose of COVID-19 vaccination based on the BASNEF model used a review of studies that determined the effective factors that lead to healthy behaviors [12, 24–26]. Attitude (3 items, e.g., "Getting a booster dose of coronavirus vaccine helps prevent coronavirus disease in me.”) or, "I am healthy and do not need to be vaccinated against coronavirus."), subjective norms (3 items, e.g., "My children and family believe that I should get the coronavirus vaccine."), enabling factors (3 items, e.g., "The free booster dose coronavirus vaccine increases the acceptance of this vaccine."), intention (2 items, e.g., "I plan to get the booster dose of coronavirus vaccine on time for the elderly."), These items were assessed using a 5-point Likert scale from 1 strongly disagree to 5 strongly agree and behavior of subsequent dose of COVID-19 uptake (2 items, e.g., "I get the booster dose of coronavirus vaccine on time."), These items were assessed using a 5-point Likert scale from 1 strongly disagree to 5 strongly agree. The range scores of each subscale are presented in Table 2.

Content validity ratio (CVR) and content validity index (CVI) were used to determine content validity. Ten experts with health education, health promotions, and epidemiology immunology specialists checked it. The result showed high overall CVI and CVR on the scale. The mean scores for the CVI and CVR were 0.92 and 0.80, respectively.

### Data analysis and validity assessment

**Fig. 3:**
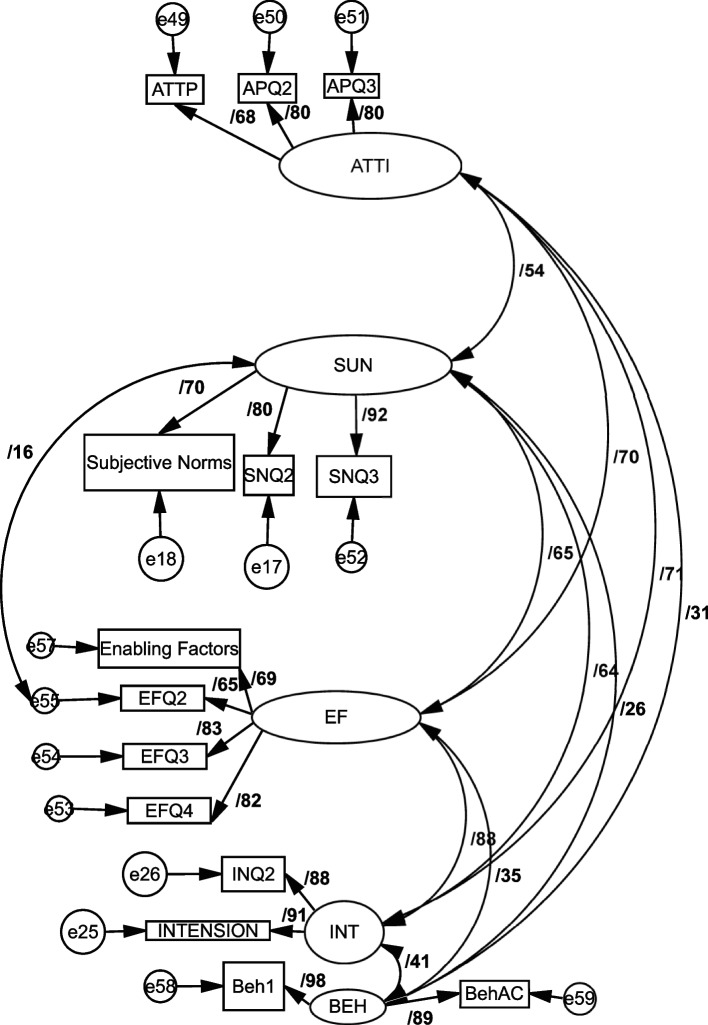
Confirmatory factor analysis (CFA)

The two-step process is adopted for analyzing data. The first step, Confirmatory Factor Analysis (CFA), uses the software IBM AMOS-24. Average variance explained (AVE), construct reliability (CR), and maximum shared variance (MSV) are computed for all factors and are presented in Table 3. The average variance extracted (AVE) values were higher than 0.5 [27] Tables 3, 4; Fig. 3. The initial model is generated for five constructs, and outcomes are used to analyze the model's goodness of fit and construct validity.

In the second step, structural equation modeling (SEM) is carried out to analyze the proposed model's path. The effect of relationships amongst the theoretical constructs is also analyzed using SEM. Structural Equation Modeling (SEM) is a technique used to specify, estimate, and evaluate linear models among a set of observed variables in terms of an often smaller number of unobserved variables. SEM may be used to build or test the theory. The one-way analysis of variance (ANOVA) and independent sample T-test were used to analyze the data with SPSS version 23.

## Results

### Descriptive statistics

The age range of the study participants was between 60 and 97 years, with a mean age (± SD) of 66.77 ± 6.26 years, and other demographic information was mentioned in Table 1.

**Table 1:** Association between intention to booster dose of COVID-19 vaccination and demographic variables (*n* = 1120)

Factor	Number	Per cent	Mean ± SD	*p*^a^
**Age (yrs.)**				
60–64	421	37.6	8.42 ± 1.68	0.001^**^
65–69	410	36.6	7.66 ± 2.43	
70–74	164	16.6	7.73 ± 2.16	
75–79	57	5.1	7.72 ± 2.08	
≥ 80	68	61	7.22 ± 1.86	
**Gender**				
Female	553	49.4	7.61 ± 2.20	0.001^**^
Male	567	50.6	8.25 ± 1.99	
**Educational level**				
Illiterate	339	30.9	6.96 ± 2.47	0.001^**^
Under Diploma	109	9.7	7.69 ± 2.28	
Diploma	228	20.4	8.45 ± 1.59	
Upper Diploma	444	39.6	8.47 ± 1.71	
**Marital status**				
Married	756	67.5	8.21 ± 2.12	0.001^**^
single	168	15.0	7.73 ± 1.98	
Widow	76	6.8	6.97 ± 2.29	
Divorced	33	2.9	6.97 ± 1.61	
Partner dies	87	7.8	7.16 ± 1.82	
**Number of families**				
**1**	109	9.7	7.64 ± 2.03	0.020^*^
**2**	316	28.2	7.73 ± 2.38	
** ≥ 3**	695	62.1	8.07 ± 1.99	
**Economic Status**				
Poor	214	19.1	7.37 ± 3.32	0.001^**^
Medium	688	61.4	8.38 ± 1.86	
Good	218	19.5	7.08 ± 2.29	
**Area of residence**				
Rural area	947	84.6	8.11 ± 2.04	0.001^**^
Urban area	173	15.4	6.94 ± 2.25	
**Chronic condition of disease**				
Yes	965	86.2	8.12 ± 1.92	0.001^**^
Not reported	155	13.8	6.75 ± 2.83	
**Diabetes**				
Yes	963	86.0	7.91 ± 2.04	0.806
Not reported	157	14.0	7.94 ± 2.13	
**Hypertension**				
Yes	869	77.6	7.87 ± 2.18	0.063
Not reported	251	22.4	8.15 ± 1.89	
**Chronic heart disease**				
Yes	966	13.8	7.92 ± 2.15	0.501
Not reported	154	86.3	8.04 ± 1.90	
**Chronic lung disease other than asthma**				
Yes	117	10.4	7.56 ± 1.79	0.047
Not reported	1003	89.6	7.98 ± 2.15	
**Had a positive test for COVID-19 infection**				
Yes	367	32.8	7.66 ± 2.07	0.001^**^
Not reported	753	67.2	8.06 ± 2.13	
**Knows someone in the family who had COVID-19**				
Yes	490	43.8	7.98 ± 1.93	0.473
Not reported	630	56.3	7.89 ± 2.25	
**Influenza vaccine uptake**				
Yes	410	36.6	7.77 ± 2.42	0.056
Not reported	710	63.4	8.02 ± 1.92	

^a^Test of significance based on the one-way ANOVA OR independent T-test

*Significant at the 0.05 level. **Significant at the 0.01 level

Besides, there was a significant association between the vaccination intention and age, sex, living with others, economic status, region and area of residence, chronic condition of disease, and a positive test for COVID-19 infection (*P* < 0.01).

Moreover, Older age ≥ 80 was associated with weaker vaccination intention than those who had a younger generation (mean = 7.22 ± 1.86 vs 8.42 ± 1.68; *p* < 0.001). A male was associated with stronger vaccination intention than a female (mean = 8.25 ± 1.99 vs 7.61 ± 2.20; *p* < 0.001). Upper Diploma individuals had stronger vaccination intentions than illiterate individuals (mean = 8.47 ± 1.71 vs 6.96 ± 2.47; *p* < 0.001). Living with others and the number of families ≥ 3 was associated with stronger vaccination intention than the individual with family = 1 (mean = 8.07 ± 1.99 vs 7.64 ± 2.03; *p* < 0.001).

High-socioeconomic status (SES) individuals had significantly higher vaccination intention scores than those who had low or medium-socioeconomic status (SES) (mean = 7.08 ± 2.29 vs 7.37 ± 3.32; *p* < 0.001).

Divorce and widow persons (mean = 6.97 ± 2.29, 6.97 ± 1.61) had significantly lower vaccination intention scores than married (mean = 8.21 ± 2.12; *p* < 0.001). However, individuals who lived in urban areas had lower vaccination intention scores than those who lived in rural areas (mean = 6.94 ± 2.25 vs. 8.11 ± 2.04; *p* < 0.001). Also, Individuals with the chronic condition of disease (mean = 8.12 ± 1.92) had higher vaccination intention scores than those who had not reported any chronic disease condition (mean 6.75 ± 2.83; *p* < 0.001). Although, individuals who had a positive test for COVID-19 infection had lower vaccination intention scores than those who had not reported any COVID-19 infection condition (mean ± SD = 7.66 ± 2.07 vs 8.06 ± 2.13; *p* < 0.001) Table 1**.**

Although the mean score of the cognitive impairment test for 6-CIT in males was 7.50 and in females was 7.33.

Also, the mean score of IADL in males was 11.1, and females was 13.01. So, nobody of the individuals had any cognitive impairments.

### Descriptive statistics of items in the BASNEF Model

It was relatively desirable. The values obtained for the structures of the BASNEF model among the participants in the study show that among the constructs of the studied model, vaccination knowledge, with 78.25% of the average score of the maximum achievable score, has the highest frequency and status. It is necessary to explain that this percentage is a kind of correct judgment and the mean alone cannot be judged and how to calculate it as the ratio of the difference between the mean of the minimum score on the range of scores is expressed as a percentage.

Also, the structures of the enabling factors were evaluated with 65.69% of the mean score of the maximum achievable score in a favorable situation respectively. At the same time, the attitude was evaluated with 69.2% of the mean score of the maximum achievable score in a relatively favorable situation Table 2. Notably, the structures of the enabling factors were evaluated with 75.69% of the mean score of the maximum achievable score in a favorable situation rather than the structure of behavior with 20.88%, which had lower the mean score of the maximum achievable score in all structures.

**Table 2:** Mean of BASNEF variables

Construct	mean (SD)	No. Questions	Range	Percentage^**a**^
Attitude	11.29 ± 2.44	3	3–15	69.08
Subjective Norms	11.95 ± 2.45	3	3–15	74.58
Enabling Factors	16.11 ± 3.21	4	4–20	75.69
Intention	7.93 ± 2.12	2	2–10	74.13
Behavior	3.67 ± 1.40	2	2–10	20.88

^a^Percentage of the mean from the maximum obtainable score

Also, the mean score of the maximum achievable score is a kind of correct judgment as a percentage. So, the mean alone cannot be judged, and it is calculated as the ratio of the difference between the mean of the minimum score on the range of scores, which is expressed as a percentage.

### Confirmatory composite analysis

The standardized loadings values were higher than 0.7 [28], with a t-statistic above ± 1.96. The composite reliability values were ≥ 70. The values of average variance extracted (AVE) presented were higher than 0.5 Tables 3, 4; Fig. 3.

**Table 3:** Convergent validity results assure acceptable values (Factor loading, Convergent validity, Discriminant validity, and reliability ≥ 0.70 & AVE > 0.5)

Construct	Items	Item Loadings	CR	AVE	MSV	MaxR(H)
Attitude	AP1 AP2 AP3	0.679 0.800 0.800	0.805	0.581	0.510	0.815
Subjective Norms	SQ1 SQ2 SQ3	0.699 0.798 0.922	0.851	0.658	0.427	0.894
Enabling Factors	EF1 EF2 EF3 EF4	0.692 0.653 0.835 0.822	0.839	0.569	0.769	0.858
Coronavirus vaccination intention	INT1 INT2	0.909 0.876	0.887	0.797	0.769	0.890
Behavior	Beh1 Beh2	0.982 0.893	0.937	0.881	0.166	0.969

**Table 4:** Correlations among constructs by off-diagonal values

	**Subjective Norms**	**Vaccination intention**	**Attitude**	**Enabling Factors**	**Behavior**
**Subjective Norms Attitude**	**0.811**				
**Vaccination intention**	0.639^**^	**0.893**			
**Attitude**	0.540^**^	0.714^**^	**0.762**		
**Enabling Factors**	0.654**	0.877^**^	0.705^**^	**0.755**	
**Behavior**	0.259**	0.407**	0.313^**^	0.349^**^	**0.939**

**Significance of Correlations: *p* < 0.001

Two types of validity are carried out for construct validity: convergent and discriminant. Average variance explained (AVE), construct reliability (CR), and maximum shared variance (MSV) are computed for all factors and are presented in Table 3. The AVE for each construct should be greater than 0.50; CR should be more than 0.7, and CR is expected to be greater than AVE [29]. Results of the proposed model fulfils the convergent validity.

To check the discriminant validity, the MSV was compared with AVE, and the square root of each dimension's AVE was compared with the correlations for each pair of dimensions addressed by AVE and MSV (AVE > MSV) as presented in the correlation matrix Table 3; the MSV of all factors was lower than AVE, except Enabling factors, which might be because of the low number of items (4 items of factor).

Based on the discriminant validity evidence, the square root value of AVE was greater than the correlation values among the latent variables [30] Table 4.

According to the GOF (goodness-of-fit) indices, the studied model fits appropriately to the standard accept one database [31]. Thus, the Confirmatory Factor Analysis (CFA) proves the model's adequacy and the decent fitting of its structural model for the participants. Table 5 represents the model fit indices.

**Table 5:** Measurement model-fit index

Measure	Recommended value	Result Value	Remark
Chi-square/degree of freedom	< 3	2.925	Good fit
Tucker Lewis Index	> 0.9	0.982	Good fit
Comparative Fit Index	> 0.9	0.987	Good fit
Goodness of Fit Index (GFI)	> 0.9	0.977	Good fit
Root mean square error of approximation	< 0.1	0.041	Good fit

This study also examined the hetero-trait-monotrait (HTMT) ratio of the correlations; thus, it re-Confirmed the presence of discriminant validity across the constructs. The HTMT values were considered lower than 0.9 [27] Table 6**.**

**Table 6:** Heterotrait-monotrait ratio (HTMT)

	**Subjective Norms**	**vaccination intention**	**Attitude**	**Enabling Factors**	**Behavior**
**Subjective Norms**	-				
**Vaccination intention**	0.665	-			
**Attitude**	0.561	0.718	-		
**Enabling Factors**	0.728	0.874	0.708	-	
**Behavior**	0.256	0.404	0.327	0.352	-

### Assessment of structural model (Inner model)

**Fig. 4:**
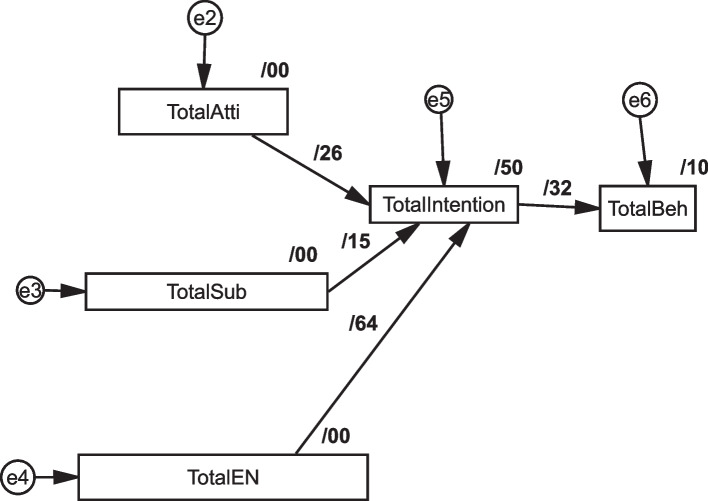
Path coefficient

The AMOS revealed the model's predictive power regarding endogenous latent variables' coefficient of determination (R2). Presented the results of analysis, the model 50% of the variance of vaccination intention as the dependent variable (R square = 0.497) and 10% of the variance of behavior as the dependent variable (R square = 0.104) can be explained based on the BASNEF model Table 7; Fig. 4.

**Table 7:** Results of structural model

**Relationship**	**R**^**2**^**(Path** coefficient**)**	***p*-value**	**Direction**	**Decision**
H1: Attitude—> vaccination intention	**0.265**	**0.000**	**Positive**	Supported**
H2: Subjective Norms—> vaccination intention	**0.148**	**0.000**	**Positive**	Supported**
H3: Enabling Factors—> vaccination intention	**0.636**	**0.000**	**Positive**	Supported**
H4: vaccination intention- > Behaviour	**0.322**	**0.000**	**Positive**	Supported**

**Research Hypotheses Significant at p** < 0.01, p* < 0.05)

### Structural model analysis

**Fig. 5:**
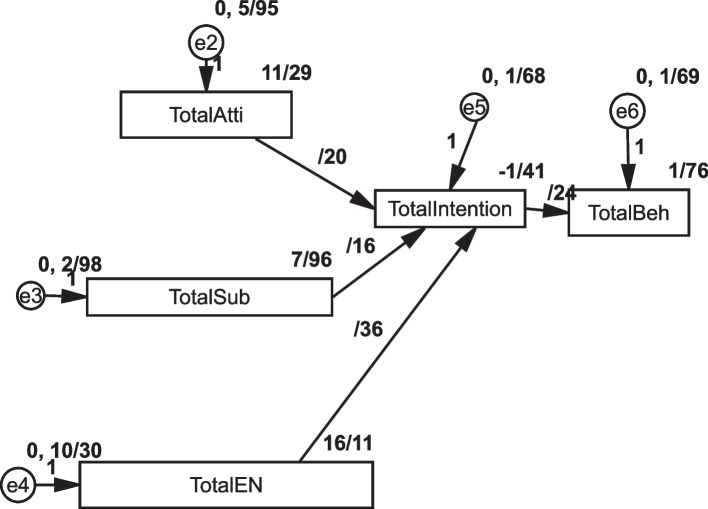
The result of bootstrapping to check the mediation effect

H1 hypothesizes show that based on the result, attitude (β = 0.265, t-value = 11.29, *p* < 0.001) is positively associated with vaccination intention. Also, H2 hypothesizes that released subjective norms (β = 0.148, t-value = 7.96, *p* < 0.001) were positively associated with vaccination intention. H3 hypothesized showed positive associations between enabling factors norms and intention (*p* < 0.001). Moreover, H4 hypotheses indicated it was associated with intention with behavior (*p* < 0.001) Table 7; Figs. 4 and 5.

### Mediation analysis

The study assessed the mediating role of intention on the relationship between attitude, subjective norms, and enabling factors on behavior. The results revealed a significant indirect effect of the impact of attitude on behavior 085 (95% CI: 0.066 ~ 0.112), subjective norms 048 (95% CI: 0.028 ~ 0.067) and enabling factors on behavior 205 (95% CI: 0.171 ~ 0.247) were positive and significant, supporting H5, H6, H7. Furthermore, the direct effect of attitude on intention (b = 0.199, *p* = 0.000) subjective norm on intention (b = 0.157, *p* = 0.001) and enabling factor on intention in the presence of the mediator was also found significant (b = 0.363, *p* = 0.001). Hence, intention partially mediated the relationship between attitude, subjective norms, enabling factors, and behavior. The mediation analysis summary is presented in Table 8; Fig. 5.

**Table 8:** Results of mediation analysis

Relationship	Direct Effect	Indirect Effect	Confidence Interval		*P*-value	Conclusion
			Lower Bound	Upper Bound		
H5: Attitude—> intention- > vaccination behaviour	0.199 (0.001)	0.085	0.066	0.112	0.001	Partially Mediation
H6: subjective norms—> intention- > vaccination behaviour	0.157 (0.001)	0.048	0.028	0.067	0.003	
H7: Enabling factors—> intention- > vaccination behaviour	0.363 (0.001)	0.205	0.171	0.247	0.001

## Discussion

The current study utilized a BASNEF Model framework to identify Factors influencing subsequent doses of COVID-19 vaccination among older adults, which, in line with Kan et al.'s study, showed that the theories of intention-oriented such as TRA and BASNEF, applicable to predicting the behaviour-related factors influencing influenza vaccination among older adults [5]. Although all BASNEF model constructs were considered desirable, Contrary to Ruiz et al. study, COVID-19 vaccination intentions were weak [32]; we can mention the different socio-demographic characteristics of the target group of the studies and the population size may lead to differences in the results.

Based on the results, 50% of the variance of intention can be explained by the BASNEF model. Also, the results of Griffin et al. reported that the PMT (protective motivation theory) explained 59% of the variance in COVID-19 vaccination intentions, which is in line with the present study [33]. All variables of the BASNEF model were significant predictors. So, with planning and implementation, vaccine promotional activities could improve COVID-19 vaccine uptake in older adults.

While, based on the present finding, older adults aged > 80 years seemed to have a lower vaccination intention than other age groups, in line with this result in Lau et al.'s study, individuals aged > 80 years were less probably vaccinated intention than younger ages [34]. Also, Kan et al.'s study mentioned that age was one factor of influenza vaccination [1]. It seems that loneliness experiences with not sufficient social support and cognitive problems in prospective memory refer to the task of remembering to perform intended actions after a delay without an explicit reminder, such as remembering to take medication on time or healthy behavior may be the cause of lower intentional activity in an old-old age group (80–91 years) [35].

According to the findings, vaccination intention is associated with sex; in elderly males, vaccination intention was more than in females.

In line with this result, the study of Mangtani et al. indicated that vaccination intention was higher among elderly males than elderly females [36]. The current findings also align with meta-analyses by Zintel et al., which noted that significantly fewer women would get vaccinated than men, OR 1.41 (95% CI 1.28 to 1.55) [37]. Moreover, Kan et al.'s study mentioned that one factor of influenza vaccination was sex [5]. Lower vaccination intentions among women could be problematic because women have a central role in ensuring the health of their families.

Besides, vaccination intention is associated with living with others; similarity, Kan et al.'s study mentioned one factor of influenza vaccination was living with others [1]. Older adults who lived with family were more likely to have been vaccinated [38]. This finding highlighted the role of social support for all healthy intentional behavior.

Based on the present study, Ruiz et al.'s study, being college-educated and married people were all associated with stronger COVID-19 vaccine intentions [2], which aligns with the current study. Generally, having a partner or supportive family member was associated with health-promoting behaviors, especially during the COVID-19 pandemic [39, 40].

This finding indicates that having a positive test due to COVID-19 infection was significantly correlated with COVID-19 vaccination intentions and uptake. Such a finding is also consistent with research on health behavior, indicating that past behavior strongly predicts future behavior [41].

The current study showed that uptake of an influenza vaccination was not significantly correlated with COVID-19 vaccination intentions and uptake. Similarly, the size of the correlations between the uptake of influenza vaccination and COVID-19 vaccination intentions and subsequent uptake were small in Griffin et al.'s [33].

Moreover, other findings showed that attitude was associated with intention. Some of the impact of beliefs and attitudes (e.g., concerns about the safety of COVID-19 vaccines) on intentions to be vaccinated may be linked to trust issues [33]. Lin et al. highlighted trust as an important factor concerning people's decisions about whether or not to be vaccinated [42]. Mostly knowledge-sharing behavior, correcting wrong beliefs and superstitions is essential to improving behavioral intention [34]. Also, inviting vaccinated older adults to share their positive experiences of vaccination in retirement or other older adults' gathering centers was suggested.

Based on the results, enabling factors were the best predictors of vaccine intention and subsequent uptake. Similar findings have recently been reported by Hsieh et al.; the enabling factor was counted as one factor associated with using adult preventive health services [43]. Duval et al.'s study indicated that several modifiable factors, including knowledge, perceived self-efficacy, and societal and colleagues' support, were associated with willingness to recommend vaccines [44]. Generally, coping appraisals and implementation of improving coping strategies, such as promoting self-efficacy in older adults, cause more robust correlates of protective intentions that intention is the strongest correlate of future behavior [45]. So, These results emphasize the importance of improving motivation and enabling factors for vaccination and handling distrust [46].

Based on the results, intention was the best predictor of vaccine behavior and subsequent uptake. These findings align with growing evidence that in the reason-based approaches, the stronger the intention, the more likely the behavior will follow [47].

The current study has several strengths. First, the study focused on the intention and behavior of the subsequent doses of COVID-19 vaccines instead of solely considering the intended uptake, which has been the focus of almost all research to date. Second, applying a theoretical perspective, the current findings indicate that the reason-based approaches, such as the BASNEF model, provide an appropriate theoretical framework for considering the determinants of Covid-19 vaccination intentions and uptake. Also, the cognitive impairment tests were applied to the present study with the target group of older adults.

This study has several limitations. First, since this was a cross-sectional study, identifying additional factors in future research was recommended. Second, the potential for interviewer biases may be included, and which longitude study design could help manage bias. However, the findings of this study might not be generalized to all populations of older people. Therefore, future research can investigate factors influencing intention to COVID-19 vaccination uptake from a more behavioral approach by a broader population of individuals in specific cultural backgrounds.

## Conclusion

The present study demonstrates that planning and implementing promotional intervention programs for older people (over 65; 80), females, illiterate, widows, and divorced people with good economic status and urban areas is essential. It seems that enabling factors such as free vaccinations, vaccination inaccessible places such as public social security agencies, social supports such as involvement of the government and physicians, and improving information about the COVID-19 vaccine by the medium which can be further used to enhance the acceptance of subsequent doses of COVID-19 vaccine uptake in older adults. Moreover, vaccination campaigns or peer education strategies for knowledge sharing might boost vaccination intention and uptake among older adults and can be suggested to stakeholders or other educators in this field.

## Data Availability

The datasets used and analyzed during the current study are available from the corresponding author at reasonable request.

## Abbreviations

BASNEF Model: (Beliefs, attitudes, subjective norms, and enabling factors) Model
TRA: Theory of Reasoned Action
HBM: Health Belief Model
CVR: Content validity ratio
CVI: Content validity index
SEM: Structural Equation Modeling
CFA: The Confirmatory Factor Analysis
AVE: Average variance extracted
HTMT: Heterotrait-Monotrait Ratio
